# Amodal Completion Revisited

**DOI:** 10.1177/2041669520937323

**Published:** 2020-08-30

**Authors:** Walter Gerbino

**Affiliations:** Department of Life Sciences, Psychology Unit Gaetano Kanizsa, University of Trieste, Italy

**Keywords:** amodal presence, occlusion, good continuation, visual interpolation, filling in

## Abstract

Amodal completion (AC) is analyzed, by looking at its historical roots and persisting conceptual difficulties. Looking at the origin of the concept, it becomes clear that it is not equivalent to perception of occluded parts. The role of fragment incompleteness is discussed, to clarify that it cannot be taken as a necessary factor for eliciting AC. The standard view of AC, depicted as a set of processes that extrapolate from veridically represented image fragments, is evaluated and rejected on the basis of evidence that AC modifies also modal parts. The theoretical importance of AC phenomena and their potential to reveal the inner forces of perceptual organization are emphasized, with specific reference to the minimum principle. Instances in which AC might be expected but does not occur are examined, to define the limits of such an integrative process.

Amodal completion (AC) has grown in interest—as demonstrated also by this special issue of *i-Perception*—but has kept the enigmatic nature that marked its official birth in the scientific literature (Michotte & Burke, 1951/1962), possibly because of persisting ambiguities in its definition and the intrinsic vagueness of phenomena grouped under its umbrella.

The recent literature on AC includes, besides phenomenological and psychophysical studies (among others Ekroll et al., 2018; Øhrn et al., 2019; Peta et al., 2019; Scherzer & Ekroll, 2015; Scherzer & Faul, 2019; for a partial review, see van Lier & Gerbino, 2015), philosophical discussions that elucidate the theoretical impact of empirical research on amodal phenomena (Briscoe, 2018; Brown, 2018; Nanay, 2018; Orlandi, 2014), animal research on recognition of amodally completed shapes (Pepperberg, 2017; Pepperberg & Nakayama, 2016), as well as neural models of pattern completion (Tang & Kreiman, 2017). The fast-growing neuroscientific evidence relevant to AC and related processes has been reviewed by Thielen et al. (2019). As regards artificial vision, some computational models embody AC processes (Follman et al., 2018; Oliver et al., 2016; Zhu et al., 2017), while occasional references to AC are found in the broader literature on object recovery from images with missing regions, not necessarily due to occlusion (Ehsani et al., 2017; Guo et al., 2018; Han et al., 2017; Harary et al., 2014; Hueting et al., 2017; Li & Malik, 2016; Mavridis et al., 2015; Oliver et al., 2017; Pérez et al., 2016; Shao et al., 2014).

To favor the organization of such a diverse literature within a consistent framework, the present article presents a brief history of contributions that shaped the AC concept as currently used and discusses a selected set of issues that underlie a large proportion of the flourishing AC research field.

## History

Fundamental ideas about AC, as well as the awareness of its relevance to perceptual theory, precede Michotte’s and Kanizsa’s coinage of the label in the 1950s. Husserl (Almäng, 2014; Madary, 2017, p. 108) qualified the phenomenal presence of occluded portions of external objects as a perceptual anticipation. Koffka (1935, p. 178) used “representation without color” to define the feeling of smooth continuity of the ground in the region of the visual field perceived as figure; he also critically analyzed the context-sensitive meanings of “to see” in his famous excursion in a law court, where a witness is cross-examined about a revolver possibly concealed under a book lying on the table (Koffka, 1935, p. 180).^1^

Metzger (2006, Chapter 8) emphasized that the “invisibly present” (*unsichtbar Vorhanden*) is a ubiquitous component of visual experience, which apparently compensates for optic occlusion; like Koffka, he discussed the law court as an unnatural setting where one is forced “to separate in his observations what certainly was perceived from what was only suspected” (Metzger, 2006, footnote 73, p. 134). Representation without color and invisible presence are appropriate labels for phenomena that can be interpreted as instances of a pervasive bias at the interface of perception and cognition, imaginatively named the “etcetera principle” by Gombrich (1960, pp. 185–186).

### Michotte and Burke (1951), Burke (1952), and Glynn (1954)

The effective starting point for empirical research on AC was a talk delivered at the XIII International Congress of Psychology, held in Stockholm in 1951, and summarized in a short paper on “a new enigma in the psychology of perception” (Michotte & Burke, 1951/1962). Despite its conciseness, this article outlined a systematic approach to stimulus conditions and possible endogenous determinants of amodal phenomena. Interestingly, Michotte and Burke (1951/1962) did not use the AC label explicitly. Rather, they introduced the phenomenological notion of “amodal datum” (*donné amodal*) to capture a general feature of perception; that is, the fact that the world experienced by human observers includes modal and amodal parts. Referring to vision, they claimed that the world, besides the directly visible modal parts—so named because they are locally marked by the quality characteristic of the visual modality (i.e., color)—also contains amodal data: “invisible” parts taken as real despite the absence of local color.

Michotte and Burke (1951/1962) discussed two paradigmatic instances of amodal presence: the tunnel effect (involving the continuous presence of a moving object behind a screen, when only the entry and exit motion segments are modally visible) and the perceived back of opaque solid bodies (involving the colorless continuation of the front surface into the occluded space). At the end of their paper, while discussing this second instance, they evoked the notion of completion (conceived as a fact, not as a process):

apparently, the “visible” part is *completed* by the “amodal” presence of the posterior part of bodies and such presence, as well as the properties that characterize it, are determined by the structure of the “modal” datum and, in the last analysis, by the system of visual excitations. (Michotte & Burke, 1951/1962; my translation and italics)

The modal/amodal phenomenological dichotomy, as used by Michotte and Burke (1951/1962), differentiates two types of visual data: with versus without color. In most contemporary literature (van Lier & Gerbino, 2015), the modal/amodal contrast is rather used to differentiate two types of completion that share, as a common psychophysical trait, the lack of a local stimulus counterpart. In their phenomenological analysis, Michotte and Burke disregarded illusory modal completions (MCs) and considered only modal parts with a local stimulus counterpart, upon which also amodal data eventually depend.

In his paper on the tunnel effect, Burke (1952) suggested that “the absence of sensory qualities justifies the use of the term ‘amodal data’ to describe the way in which the hidden movement phase makes itself known to the observer” but did not use the AC label either. He claimed that, under optimal conditions for spatiotemporal continuity, the amodal phase acts as a “bridge” between the modal phases, becoming an integral part of the total sensory experience.

Instead, the term “completion” was frequently used by Glynn (1954) but to refer to the modal—not amodal—presence of the perceptually interpolated portion of a moving object in conditions originally described by Rosenbach (1902; Metzger, 1936/2006, Figure 141). Under Michotte’s supervision, Glynn made a systematic investigation of the Rosenbach phenomenon, known as “visual phantoms” in the more recent literature (Maguire & Brown, 1987; Kitaoka, Gyoba, Kawabata, et al., 2001; Kitaoka, Gyoba, Sakurai, et al., 2001; Tynan & Sekuler, 1975).

Concerning a possible relationship between modal and amodal ways of bridging the spatiotemporal gaps due to optic occlusion, Glynn (1954) formulated his own “identity hypothesis”—to borrow a term from Kellman and Shipley (1991; Shipley & Kellman, 1992)—as follows:

The “modal presence” of the covered parts in Rosenbach’s experiment and their “amodal presence” in the “screen effect” and “tunnel effect” suggested that the two might differ only in degree and not in kind. If such a continuity could be established, the “amodal presence” of the covered parts in the latter cases could eventually be considered as the lowest limit of the apparent transparency, corresponding to a system of excitation inferior to its threshold. (p. 126)

Experimental evidence led Glynn to reject the hypothesis of a continuity between the Rosenbach phenomenon and the tunnel effect, and to label Rosenbach’s apparent transparency but not Burke’s hidden perceived motion as “figural (or form) completion (or continuity).”

### Kanizsa (1954, 1955/1987)

In the same year, Kanizsa (1954) explicitly used the expression “completamento amodale” (AC) for a process that—according to his proposal—would constitute a necessary, though not sufficient, condition for the formation of illusory contours. This feature of the AC concept was elaborated in his extensive paper on “quasi-perceptual margins” (Kanizsa, 1955/1987). Two aspects of Kanizsa’s theorizing were original: (a) first, the AC label was used not only for a set of *phenomena* (e.g., perceiving three black pacmen as disks and three chevrons as a closed outline triangle, partially occluded by the illusory triangle) but also for a *process* instantiated by the autochthonous tendency to form improvement and (b) second, the modal presence of illusory occluders was explained as a product of AC, driven by form improvement.

In other terms, Kanizsa (1954, 1955/1987) proposed a “causal hypothesis” (van Lier & Gerbino, 2015) in which AC explained at least some instances of MC such as the illusory triangle. He hypothesized that the AC process is initiated by phenomenal incompleteness, taken as the basic requirement for form improvement (an idea rooted in the dynamic model of object formation put forward by Gestalt theory). Kanizsa’s demonstrations that phenomenal incompleteness is actually necessary for AC, which eventually accounts for the emergence of a modal illusory occluder, were based on the manipulation of inducers. For instance, octagons with collinear notches supported a vivid illusory rectangle, while collinear crosses (obtained by cutting the same notched octagons symmetrically) did not. The controversial relationship between fragment incompleteness and AC will be discussed in the section “Incompleteness and AC.”

### Michotte, Thinés, and Crabbè (1964/1991)

**Figure 1.**
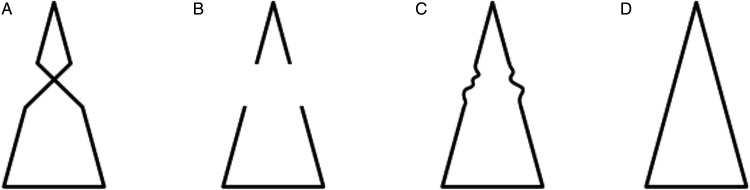
Michotte’s Triangle. Use a pencil to cover the central part of any of the four patterns (or of all patterns simultaneously). Each will look as a complete isosceles triangle. Patterns A and B were discussed by Michotte et al. (1991, Figure 3.7, p. 146, and Figure 3.3, p. 145).

The landmark essay by Michotte et al. (1964/1991) provided a systematic analysis of completion phenomena in vision and anticipated future developments in the field. As regards terminology, van Lier and Gerbino (2015, footnote 2, p. 304) noticed that the translation of *compléments amodaux* as “amodal completions” rather than “amodal complements” may sound inappropriate.^2^ However, it should be recognized that “completion” for *complément* has been chosen by the editors of the English translation of Michotte’s papers (Thinés et al., 1991) and is largely prevalent—with the notable exception of Jackendoff (1992, pp. 163–164)—even when the intended meaning is just “perceptually added parts,” without any reference to fragment incompleteness and hypothetical completion processes. Clearly, this lack of terminological precision is a potential source of confusion, which requires a critical attitude. But let us postpone the discussion of this issue to the section “Incompleteness and AC” and consider now the following points.

a. Rather than focusing on the phenomenological specificity of AC that attracted the attention of Michotte and Burke (1951/1962)— that is, on the enigma of perceptual presence in the absence of color, taken as the modal property of vision—Michotte et al. (1964/1991) emphasized a feature that *les compléments amodaux* share with MCs: The lack of a psychophysical correspondence with local stimulation.
b. Michotte et al. (1964/1991) treated MC and AC as extremes of a continuum, referring to Koffka (1935) for completion in the blind spot and hemianopia as paradigmatic cases of MC. They also classified apparent transparency under Rosenbach’s conditions as an intermediate phenomenon.
c. Michotte et al. (1964/1991) discussed several cases of AC, with and without occluder. Beyond doubt, they did not think of AC and perception behind occluders as coextensive, contrary to common definitions of AC found in the contemporary literature.^3^ Rather, they proposed to consider the perceptual presence of an invisible immaterial substance bounded by a rotating wireframe as a kind of AC and suggested that it represents the prototypical, but not the only, amodal complement “without cover” (Michotte et al., 1991, p. 160; *complément amodal ‘à découvert’*, Michotte et al., 1964, p. 44). According to Michotte et al. (1991, p. 162), the perception of 3D space and distance between objects also fall in the category of amodal complements without cover, at least to some degree.
d. Michotte et al. (1964/1991)—like Michotte and Burke (1951/1962)—claimed that AC is a function of the overall stimulation. By this formula, they meant that AC is neither a matter of arbitrary imagination (i.e., a mental content independent of current stimulation) nor a function of local visual stimulation, which normally determines the properties of surfaces perceived as occluding. However—unlike Michotte and Burke, who concluded their short paper with a generic claim about learning and past experience as possible determinants of amodal data—they argued that, when in conflict, structural factors do prevail over empirical factors. In this respect, the fundamental demonstration by Michotte et al. (1964/1991), shown in Figure 1A, is the so-called “Michotte’s triangle” (Carrigan et al., 2016). The comparison of the four panels in Figure 1 (taken from Peta et al., 2019; for a similar version, see Vicario & Kiritani, 1999) parallels an earlier demonstration by Metzger (1936/2006), reproduced here in Figure 2. In both demonstrations (Michotte’s triangle and Metzger’s cross), knowledge of the distal object—that is, the immediate memory of the objective shape made visible/invisible by the observer herself—does not penetrate perception. For instance, when contour complexities in Figure 1A and 1C or contour gaps in Figure 1B are occluded and do not contribute to the overall stimulation any more, simplicity prevails and a regular isosceles triangle emerges in perception.

**Figure 2.**
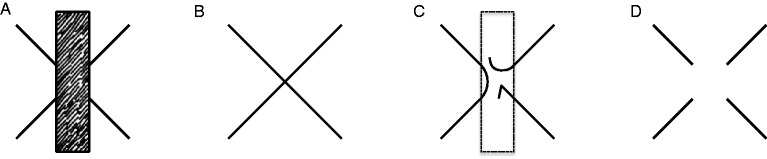
Metzger’s Cross. In Panel A, the diagonal collinear segments complete each other amodally and appear such as in Panel B. Panels C and D illustrate two (out of many) line patterns that would elicit observer’s surprise after removal of the occluding rectangle. Redrawn from Metzger (1936/2006), Figure 138.
e. In occlusion conditions, AC affects not only occluded but also unoccluded parts. Michotte et al. (1991, p. 164) based such a conclusion on the following two remarkable aspects of the tunnel effect.
1. The variation of the entry–exit time interval affects not only the perceived speed of the moving object in the central amodal phase but also in the initial and terminal modal phases, suggesting that speed is smoothly integrated (i.e., homogenized) within the whole spatiotemporal segment occupied by the event (Michotte et al., 1991, p. 153).
2. In the limiting case in which the vertex of the angle formed by entry and exit rectilinear trajectories lies exactly on one side of the screen (Burke, 1952, p. 123, Figure 1, Panel B; Michotte et al., 1991, Figure 3.9B), at least some observers reported that the coincidentally occluded entry and exit movements were perceptually joined by trajectories curved in both their amodal and modal portions: “this completion is so integrated in the whole that it encroaches sometimes on the visible sections” (Michotte et al., 1991, p. 156). The theoretical implications of this distorted perception of modal portions of the moving object’s trajectory and its relevance to current research will be evaluated in the section “AC affects modal parts too.”

**Figure 3.**
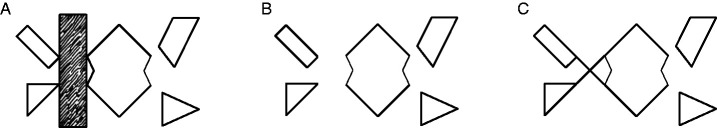
In panel A, the arms of the diagonal cross of figure 2 are embedded in closed, stable structures (Rectangle, Triangle, and Concave Octagon) and do not continue behind the dark vertical rectangle. The pattern in Panel B is perceived as the natural one after the removal of the dark vertical rectangle, while the central cross in Panel C is perceived as a surprising addition. Redrawn from Metzger (1936/2006), Figure 139.

## The Bregman–Kanizsa Effect

As highlighted by Peta et al. (2019), two AC phenomena can be considered paradigmatic: One is Michotte’s triangle shown in Figure 1 (as well as Metzger’s cross shown in Figure 2), that is a case of conflict between perception and the distal stimulus; the other is the Bregman–Kanizsa effect, that is a case of conflict between perception and the proximal stimulus.

The Bregman–Kanizsa effect is a label (introduced by Nakayama et al., 1989) for the identification gain produced by AC, with respect to a comparison situation in which conditions for the unification of input fragments (typically, the presence of an adjacent surface acting as occluder) are removed and input fragments are perceived as isolated shapes. Kanizsa (1979, Figure 1.1a vs. Figure 1.2b) illustrated this effect using a set of fragments that only thanks to added T-junctions are immediately perceived as the visible components of a partially occluded cubic structure. Bregman (1981) compared two pictures containing the same fragments, perceived either as such or as the visible parts of easily recognizable amodally completed letters. In both Kanizsa’s and Bregman’s demonstrations, the structures revealed by the addition of occluders (i.e., by AC) are recognizable—though with some effort—also in the absence of the occluder.

**Figure 4.**
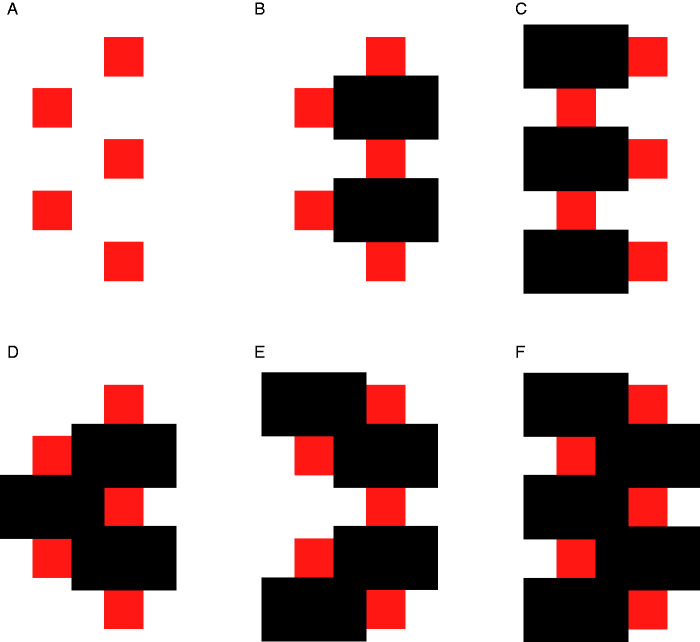
The same five squares, perceived as such in panel A, become the modal parts of different amodally completed surfaces, each with a distinctive shape. Partially occluded red objects in Panels C and F are familiar and easily recognized as the capital letter E and a vertically elongated rectangle, respectively; but also the less familiar completions in Panels B, D, and E are perceived without effort. As discussed in the text this is an extreme case of the so-called Bregman–Kanizsa effect.

However, Peta et al. (2019) emphasized that the Bregman–Kanizsa effect can be so strong that the observer cannot perceive the superordinate object revealed by the juxtaposition of an occluder at all (see their Figure 2, structurally similar to the present Figure 4). Some implications of this total lack of recognition of the superordinate object produced by AC processes are discussed in the subsection “Geometric versus perceptual incompleteness.” Peta et al. (2019) considered one implication, in particular: that is, the inadequacy of the notion of “recognition from partial information,” as used in most computational literature (Bajcsy & Tidhar, 1977; Tang & Kreiman, 2011, 2017), for the emergence of objects with a recognizable shape, made possible by the juxtaposition of an occluder.^4^

In a broad sense, the Bregman–Kanizsa effect includes both facilitatory and inhibitory consequences of AC. The emergence of an amodally completed object makes the identification of the unified object possible, with respect to the mere presence of component fragments; but, at the same time, makes the shape of individual fragments less recognizable. The strength of facilitatory and inhibitory aspects of the Bregman–Kanizsa effect has been measured in several studies, by means of different psychophysical methods (Bruno & Gerbino, 1987; Chen et al., 2009; Gerbino, 1981, 1989; Gerbino & Salmaso, 1987; Johnson & Olshausen, 2005; Murray et al., 2001; Rauschenberger et al., 2004; Sekuler & Murray, 2001; Shore & Enns, 1997; Yun et al., 2018).

The facilitatory component of the Bregman–Kanizsa effect could be considered as a kind of configural superiority effect. However, Gold (2014) provided evidence that visual completion processes involve unanticipated costs. He found that discrimination sensitivity is higher when targets are perceived as isolated fragments than as parts of a whole including either MC (Bent Bars condition) or AC (Rotating Squares and Shrinking/Expanding Squares conditions). As claimed by Gold himself, these results are a challenge for future theories of visual completion.

## Incompleteness and AC

Demonstrations that perceptual completions (either modal or amodal) depend on stimulus incompleteness go back at least to Metzger (1936/2006). In his Figures 138 and 139—reproduced here in Figures 2 and 3, respectively—Metzger compared two conditions: In Figure 2A, four oblique segments tend to continue behind the adjacent rectangle; in Figure 3A, the same segments are grouped with other segments to constitute the borderlines of closed shapes and do not tend to continue behind the adjacent rectangle. Pairs of collinear segments unify themselves and complete each other amodally only when they are isolated and the points of contact with the rectangle are perceived as line endings, while they do not unify themselves when each segment is embedded in the contour of a separate shape (Figure 3).

Here, Metzger anticipated the rhetorics of other AC-based phenomenological demonstrations—such as Michotte’s triangle (Figure 1)—that exploit observer’s surprise (or lack of surprise) as a direct source of evidence of spontaneous perceptual organization. According to Metzger, the appearing of Figure 2C or 2D after removal of the occluder is unexpected and surprising, while the appearing of Figure 2B is consistent with the amodal presence of hidden crossing lines in Figure 2A.

To argue that inducer incompleteness is a determinant of modal illusory contours, Kanizsa utilized displays with notched octagons versus crosses (Kanizsa, 1954, 1955/1987) or pacmen and angles versus trilobate butterflies (Kanizsa, 1955/1987) that paralleled Metzger’s argument based on his Figures 138 and 139, with the important difference that Metzger’s occluder was real, while Kanizsa’s was illusory. This parallelism supports a structural similarity between modal completion and AC, though distinguishable from the one implied by Kellman’s identity hypothesis; that is, by “the idea that a common contour interpolation process underlies partly occluded and illusory contours,” to quote Kellman et al. (2001). The hypothesis that incompleteness is a condition for completion receives some support from the fact that its elimination by figural means (i.e., the presence of complete fragments) abolishes AC in both Metzger’s and Kanizsa’s displays. According to the causal hypothesis, in Kanizsa’s displays fragment completeness also prevents the emergence of a modal illusory occluder. However, this is not always the case. As discussed in the following subsections and demonstrated by Figure 4, complete fragments are fully compatible with AC, when some supporting conditions hold.

Interestingly, minimal conditions for the induction of an illusory blob (Ehrenstein, 1941/1987) are satisfied by Metzger’s outline segments in Figure 2D but not in Figure 3B, where they are embedded in closed contours. Metzger did not discuss this effect, which Kanizsa instead exploited in many demonstrations where line endings induce illusory contours (for instance, Kanizsa, 1979, Figures 1.1a vs. 1.13 and Figures 2.10 vs. 2.11).

Studies on the role of incompleteness as a determinant of MC and AC have been reviewed by Lesher (1995) and van Lier and Gerbino (2015). The logic behind incompleteness as a determinant of illusory figures (i.e., MC) has been criticized by Pinna and Grossberg (2006). In general, the following problems related to this idea persist.

### Fragment Incompleteness

Within the domain of MC, evidence that incomplete fragments induce illusory objects (possibly, by means of AC), while comparable complete fragments do not, is logically compatible with evidence that, in different circumstances, illusory objects can be induced by elements that—when seen in isolation—look complete and, therefore, should not activate a tendency toward completion (Rock & Anson, 1979). The class of illusory objects is heterogeneous and includes perceptual entities with different properties and possibly diverse determinants. Inducer incompleteness might well be a determinant of a subclass of such phenomena, without necessarily being a determinant of all.

For instance, pacmen are individually perceived as notched disks—that is, as simpler shapes with a missing portion (Kanizsa, 1954, 1955/1987)—while rectangles and trapezoids are individually perceived as complete. Nevertheless, both can induce illusory figures and amodally continue behind them, with a degree of evidence that ultimately depends on figural factors such as parallelism (Albert, 1993).

### Geometric Versus Perceptual Incompleteness

The distinction between geometric and perceptual incompleteness is subtle and controversial. One could argue that the effective determinant of AC (and, consequently, MC) is geometric incompleteness of an entity in the proximal stimulus, independent of the actual perception of this property in a valid control condition. However, universal criteria for geometric completeness/incompleteness are hard—if not impossible—to define (Lesher, 1995, pp. 294–295) and, therefore, the only relevant criterion might be perceptual (Pinna & Grossberg, 2006).

However, the idea that incompleteness is only a perceptual property would run the risk of circularity if the occurrence of AC were the only criterion for defining a form as incomplete; that is, if one could label a form as incomplete only after AC, not before, so making impossible an a priori definition of inducer incompleteness as a determinant of AC. This logical difficulty is made clear in Figure 4, which shows that the very same set of square fragments is compatible with multiple amodally completed objects (Gerbino, 2016, Figure 3.16; for a similar example see Gerbino, 1997; for the general idea that AC can create different visual objects see Kanizsa & Gerbino, 1982, pp. 177–180).

The completed shapes perceived in Figure 4B–F cannot be explained by fragment incompleteness, for two reasons: (a) squares do not look incomplete and (b) the existence of five different amodally completed objects in Figure 4B–F is incompatible (or at least hard to reconcile) with the idea that AC depends on the properties of isolated fragments shown in Figure 4A. This idea is evidently wrong, though the rhetorics of the demonstration in Figure 4 aims at showing that the “same” elements can be combined in different ways, depending on the positions of occluders. In fact, the positioning of occluders modifies the assignment of border ownership and critically alters the identity of elements. To explain the emergence of five different objects in Figure 4B–F, one should consider the fundamental role of local T-junction segmentation (N. Rubin, 2001), a process that breaks the unity of the square contour, assigning different directions of occlusion to different sides of each square. Even for a highly symmetric shape such as a square, contour discontinuities are critical loci for two distinct processes: image segmentation and amodal continuation behind occluders.

**Figure 5.**
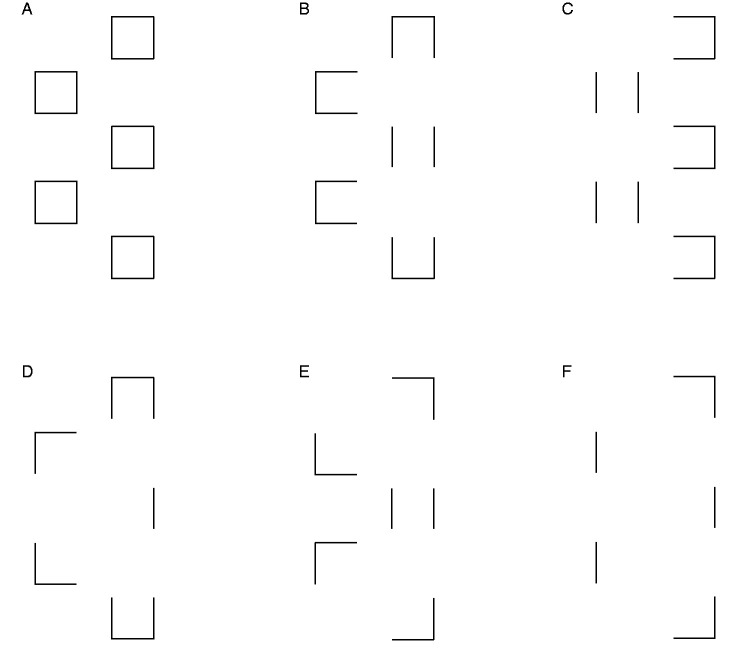
An Outline Representation of Square Regions in Figure 4. Outlines in Panels B to F represent the borders of square regions after the removal of segments belonging to borders of black occluding rectangles (i.e., after image segmentation).

As a consequence of image segmentation at T-junctions, the input to the completion process should not be represented as the set of outline squares in Figure 5A, but rather as the set of open contours in Figure 5B–F. Such easily connectable red/white contours (either collinear or perpendicular) are visually interpolated behind black occluders.

Note that consistently with the expansion of modal parts (Kanizsa, 1979, p. 190; Kanizsa & Luccio, 1978)—known also as “the occlusion illusion” (Palmer, 1999; Palmer et al., 2007; Palmer & Schloss, 2017; Scherzer & Ekroll, 2015)—the perceived amount of red increases from Figure 4B to 4F and is, however, larger than the amount of red in Figure 4A, despite the obvious geometric equivalence of the total area of the five squares in the six displays (Kanizsa & Gerbino, 1982, pp. 176–177). Although criticized by Pinna (2012), this important effect of AC on the visible amount of color—that is, on a modal aspect of object perception—is consistent with the idea that at least one component of AC processes (namely, the filling-in of surface properties within extrapolated/interpolated boundaries) is isomorphic rather than symbolic (Anstis, 2010; Komatsu, 2006; Pessoa et al., 1998; Weil & Rees, 2011). See the section entitled “AC affects modal parts too” for a further discussion of this point.

### Visual Versus Conceptual Incompleteness

To cope with intrinsic difficulties of the notion of completeness, Kanizsa (1979, pp. 18–19) distinguished two meanings of incompleteness, referred to the domains of seeing and thinking, respectively, using two examples.

In his first example, he contrasted the graphically/visually incomplete picture of a complete human body, without evidence of amputation, with the graphically/visually complete picture of an amputated man (i.e., of a conceptually incomplete human body). Only the former activates an AC process. This example contrasts the absence with the presence of interrupted outlines as markers of perceptual incompleteness. Kanizsa’s second example is more complex, involving the contrast between the graphically complete representation of a face with one eye only and the outline picture of half a face, with interrupted outlines that require a perceptual completion. The addition of an explicit occluder to the half face creates a problem, because it removes the difference between the two cases. In other words, while one can claim that the outline of the face is amodally completed, it would be inappropriate to claim the same for the fully occluded eye. Other demonstrations—such as the horse illusion (Kanizsa, 1970, Figure 13; 1979, Figure 4.17) and the cross in the checkerboard (Kanizsa, 1970, Figures 5 and 6; 1979, Figures 4.12 and 4.13)—suggest that a part is amodally represented only when it extrapolates/interpolates some input fragments. A superstructure such as a checkerboard (which is perceived as a mosaic of adjacent squares) is *not* completed when a component structure (a single square) is totally occluded; rather, the extrapolation/interpolation of interrupted contours prevails and a cross emerges. Analogously, we cannot expect perception to complete a half-occluded face by the amodal instantiation of a full eye.

**Figure 6.**
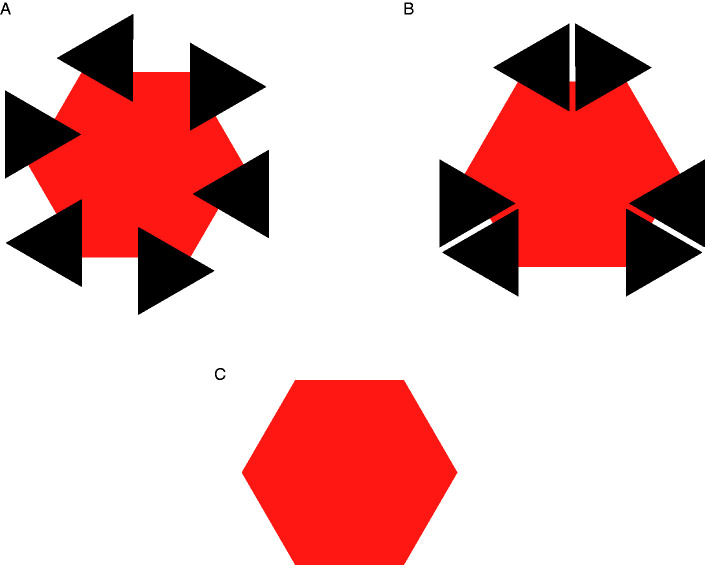
Panel A shows the Gerbino illusion in a standard display in which each angle is coincidentally occluded by a triangle whose edge is exactly superposed on its vertex. Panel B shows a variant of the illusion in which adjacent hexagon angles are occluded in opposite directions. Panel C shows a comparison unoccluded hexagon.

### What Is Completed in AC?

In principle, a part of the visual world might be taken as a “completion” only if it eliminates a gap in the proximal stimulation, or in any neural representation that literally replicates its topography. The validity of this idea heavily depends on the meaning of “gap” or—more precisely—on the shape of the entity with which the proximal stimulation is contrasted.

Two possible candidates for this entity are the distal stimulus and an object model instantiated by the proximal stimulation. This alternative corresponds to two ways of discussing AC, with reference to the partial occlusion of either (*a*) a *simple* shape such as a circle (as in many ostensive demonstrations of AC, in which observers veridically experience a circular completion behind the occluder) or (*b*) a *complex* shape such as Michotte’s triangle (leading to a peculiar case of nonveridical perception).

The first way of discussing AC (option *a*) is somewhat uninteresting, because the veridical perception of a partially occluded circle is just a coincidence. As shown in Figure 1, the same percept is obtained also when the partially occluded distal object is not a full circle. Rather, as suggested by option *b*, what matters is the object model evoked by the proximal stimulation. What is amodally filled in is a gap in this abstract entity.

Contrary to common but sloppy definitions of AC, demonstrations such as Michotte’s triangle and Metzger’s cross (Figures 1 and 2) show that the perceptual system does *not* complete the proximal stimulation in the sense of representing, though amodally, the missing projections of the distal stimulus. This faculty should be called extrasensory, not amodal, perception. Hence, definitions such as “amodal completion is the perception of hidden portions of partially occluded objects”—if taken seriously—are deeply wrong, given that they refer to irreversibly lost pieces of the distal stimulus. Rather, AC should refer to the object model evoked by information available in the proximal stimulation or, most of the times, in an early representation based on T-junction segmentation.

Probably, the physicalist implications of the term “completion” would be eliminated by translating Michotte’s *compléments* as “complements”; but the current translation has become so common in the scientific literature to discourage a terminological change. Therefore, more carefully, AC might be defined as the amodal extrapolation/interpolation of proximal parts to generate a perceptual entity consistent with the object model evoked by them. This definition includes possible cases of divergence between amodal parts and the occluded portions of the distal object, such as Michotte’s triangle and Metzger’s cross (Figures 1 and 2).

## AC Affects Modal Parts Too

The definition of AC processes as the perceptual generation of objects that include the extrapolation/interpolation of proximal parts is not totally adequate, given the evidence that the production of amodal parts also affects modal parts, and can lead to visual distortions incompatible with the hypothesis that AC interpolates input fragments, or their veridical representation (Michotte et al., 1964/1991, p. 164).

As discussed by Gerbino (2017), the difference between a completion process that leaves the representation of input fragments unchanged and one that modifies or even distorts it is captured—at least partially—by the difference between interpolation and approximation in curve fitting, given a set of points (Ullman, 1996, pp. 141–143). Interpolation is the process of generating a smooth curve that minimizes the changes of direction but connects all input points; approximation, instead, generates a smooth curve that minimizes distances from input points, without necessarily including them within its trajectory. Gerbino (2017) applied this idea to the perception of amodally completed angles in limiting occlusion conditions that lead to the so-called “Gerbino illusion” (Da Pos & Zambianchi, 1996; Gerbino, 1978). Figure 6A shows the basic effect, usually described as a distortion of the partially occluded hexagon, when adjacent sides meet at a vertex coincidentally located at the occluding edge; Figure 6B shows a variant of the illusion that enhances the perceived expansion of unoccluded modal sides; for the sake of comparison, Figure 6C shows an unoccluded hexagon.

**Figure 7.**
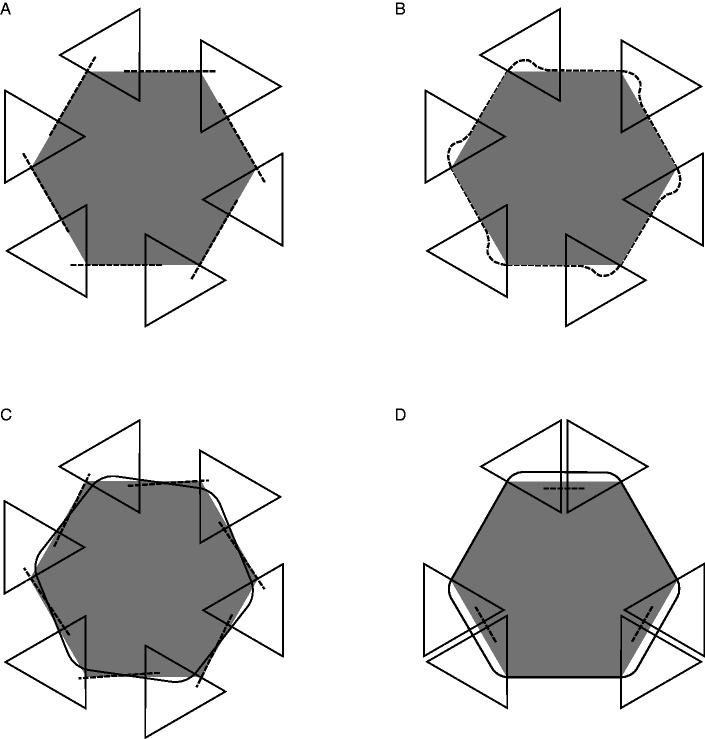
In all panels, the gray hexagons and the outline triangles represent the stimuli. The dashed outlines in Panel A represent partial completion by interpolation, constrained by good continuation only. The dashed outline in Panel B represents full completion by interpolation, constrained by smooth closure and good continuation. The rounded outline hexagons in Panels C and D illustrate completion by approximation, constrained by smooth closure and stimulus conformity, given Figure 6A and 6B as input, respectively. Within the approximation framework, the distortions perceived in Figure 6A and 6B are represented by the dashed outlines, which reflect the mismatch between the input and the approximated shapes. The dashed outlines include distorted (nonliteral) modal parts, by contrast with the internal model elicited by the coincidentally occluded hexagons.

Originally, Gerbino (1978) explained the illusion in Figure 6A within the interpolation framework. He suggested that modal sides are represented veridically and that the global perceived distortion depends on a sum of local effects in which contours exhibit an irresistible tendency to continue behind occluders after T-junction segmentation, as illustrated in Figure 7A. Here are the details of the explanation. At the contour level, each T-junction is parsed according to good continuation, assigning the two collinear T-top segments to the same unit and the residual T-stem to a different unit. At the surface level—where border ownership is established—the two T-top segments are assigned to the occluding triangle leaving the residual T-stem locally undefined but functionally disconnected from the unified T-top segments. As a result of segmentation at both contour and surface levels, T-stems continue amodally behind the occluding triangle, according to the automatic irresistible tendency to good continuation. No matter how far each T-stem continues, the final result is the loss of symmetry of the occluded hexagon.

Some observers, more sensitive to the tendency toward smooth closure, claim that the amodally present portions of the partially occluded hexagon resemble those in Figure 7B, which represents the static analog of the amodal trajectory experienced in the tunnel effect when one side of the screen lies exactly on the vertex formed by the rectilinear entry and exit trajectories (Burke, 1952, p. 123, Figure 1, Panel B; Michotte et al., 1991, Figure 3.9B).

However, independent of the degree of determinateness of amodal parts, this explanation conflicts with phenomenology, given that observers report seeing the extrapolation of the partially occluded side to intersect the modal portion of the adjacent side. Therefore, invoking good continuation is not enough. One should also assume that the vertex position is misrepresented, as if the modal portion of the hexagon side that includes the geometric vertex were perceptually expanded “beyond the vertex itself.” This is consistent with the expansion of modal parts of amodally completed shapes (Kanizsa, 1979, p. 190; Kanizsa & Luccio, 1978) as well as with an effect observed in Figure 6B. In this latter case, the unoccluded sides of the hexagon look much longer than the side of the comparison hexagon in Figure 6C, while the short visible portion of the occluded side looks too close to the hexagon center to be aligned with the virtual line connecting the extremes of adjacent long sides. For research on the phenomenal expansion of modal parts, see Vezzani (1999) and Pinna (2012), who also discussed evidence against the causal role of AC. The paradoxical nature of such expansion—which Palmer (1999) called the occlusion illusion—has been discussed by Palmer et al. (2007). Palmer and Schloss (2009) provided further evidence that the phenomenal expansion depends on occlusion and amodal continuation. For a discussion of this point and related illusions, see Palmer and Schloss (2017) and Scherzer and Ekroll (2015).

Alternatively, the illusion in Figure 6A might be explained within the approximation framework (Fantoni et al., 2007, 2008; Gerbino, 2017). In completion by approximation, proximal stimuli are not necessarily represented literally. Figure 7C shows a slightly rotated rounded hexagon that mimics (with some exaggeration due to illustration purposes) the internally generated object model accounting for the coincidental occlusion. This approximated shape includes rounded occluded angles and misoriented unoccluded sides. Angle rounding is consistent with evidence reviewed in Gerbino (2017), while the slight rotation is consistent with overarching perceptual principles such as the generic viewpoint assumption (Albert & Hoffman, 1995) and stimulus conformity (Rock, 1983, Chapter 4). Figure 6A is a limiting case of occlusion, incompatible with a generic view of a regular hexagon in the proximal orientation, but compatible with the generic view of a minimally rotated hexagon, such that its multiple-axes symmetry is preserved and conformity to the proximal stimulus is maximized. Yet, the violation of stimulus conformity, however small, has a perceptual consequence. Referring to Figure 7C, the mismatch between proximal evidence (the contour of the gray hexagon) and the internally generated shape (the black outline) explains the distortion observed in the Gerbino illusion (the dashed lines). The orientation of modal sides of the hexagon is misperceived, in the direction of contrast with the internally generated hexagon. Analogously, Figure 7D shows a representation of the approximated shape and consequent mislocation of short modal sides of the hexagon that can account for the distortion observed in Figure 6B.

Empirical results supporting the approximation approach to AC were obtained by Fantoni et al. (2007) for the 2D hexagon display and by Fantoni et al. (2008) for a 3D stereoscopic display in which a frontoparallel surface and a slanted surface were amodally completed behind a frontoparallel occluder. Previously unpublished collinearity adjustment data for the Gerbino illusion and two control conditions, consistent with approximation, are reported in the Appendix.

The above discussion about interpolated versus approximated trajectories of amodally completed contours presupposes that the distortion observed in Figure 6A depends (at least partially) on AC. This presupposition has been challenged by Pinna (2012), who aimed at showing that several effects attributed to AC are obtainable also in the absence of an occluder and, therefore, of AC (assuming that occluder presence is essential for AC). I contribute here two comments to Pinna’s arguments.

First, as noticed by Pinna himself (2012, p. 1348), in a variant of Figure 6A in which the occluding triangles are removed and only the visible segments of the outline hexagon sides are shown, lines tend to continue and induce the emergence of illusory blobs. When line endings are close enough (i.e., retinal gaps are small) such illusory blobs can be functionally equivalent to real occluders. In other words, the elimination of regions normally experienced as occluders (the black triangles in the classic Gerbino illusion) does not rule out the occurrence of AC. However, even when line endings are too far apart to induce strong illusory blobs, a tendency to unify segments into an incomplete polygon outline might be present and sufficient to produce a perceivable shape distortion, though weaker than the one produced by AC behind occluders. This possibility is related to the difference, remarked by Michotte et al. (1964/1991), between AC and mere part integration, as observed for instance in Figure 1B, where line segments available in the proximal stimulus are sufficient to evoke the presence of a triangular structure. Quantitative data on the amount of distortion observed in these three conditions (real occluders, illusory occluders, and virtual integration of contour segments without occluders) are needed to support this idea, which presupposes a continuity between different types of contour completion/integration.

**Figure 8.**
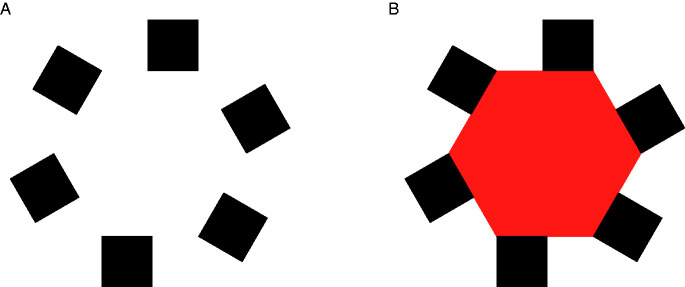
A distorted illusory hexagon can be perceived in panel A, while no comparable distortion is visible in panel B. See text for discussion.

Second, a shape distortion similar to the one attributed to AC in Figure 6A might occur in a pattern without AC, but for a different reason. This would not disprove that AC, when present, causes the distortion; it would simply show that similar effects can be produced by different causes. This observation applies, in particular, to some Pinna’s patterns (2012, Figure 9e–i) where observers report the presence of a spiral-like effect that is present also in the classic Gerbino illusion (Figure 6A) but does not constitute its distinctive aspect. As a counterargument to Pinna’s criticism, take Figure 8A, where the central illusory hexagon, when perceived, displays a distortion similar to the one in Figure 6A. Here, the distortion should be attributed to image segmentation and, in particular, to L-junctions corresponding to hexagon vertices. For global reasons, proximal squares tend to be perceived as partially occluded rectangles continuing behind a central occluder. But the segmentation of L-junction as implicit T-junctions is incompatible with an occluding hexagon with vertices in the position defined by the proximal stimulation. Hence, a distortion is perceived, with the borders between the illusory hexagon and the black background surfaces extending beyond the vertices (which is the critical aspect of the Gerbino illusion). Such a distortion is absent in Figure 8B, while the spiral-like effect, dependent on the asymmetric arrangement of black squares, is present in both panels of Figure 8.

## AC of Shape and Substance

Most research on AC has been focused on phenomena involving the emerging of unified shapes, bounded by contours intersected by occluder contours. Often contour interpolation—also due to the impact of relatability theory and the identity hypothesis (Anderson, 2007; Kellman, 2003; Kellman & Shipley, 1991; Kellman et al., 2005)—has been considered synonymous with AC. However, as remarked at the beginning of the article, AC and its conceptual antecedents include the invisible representation of surface properties, such as in the case of the table under the book, in the court apologue by Koffka (1935). Earlier, in his classic analysis, E. Rubin (1915) described the continuing of an amorphous ground behind the figure as a distinctive aspect of figure/ground articulation. Possibly, the “AC of substance” (as opposed to the “AC of shape”) is involved in the perceptual presence of the invisible volume captured by a rotating wireframe that Michotte et al. (1964/1991) discussed as the prototypical *complément amodal ‘à découvert’* (p. 44).

With the notable exception of recent work by Øhrn et al. (2019), AC of shape has attracted less attention, though it is considered in the taxonomy of perceptual experience introduced by Pessoa et al. (1998) in their influential target paper on filling-in phenomena. This taxonomy considers two orthogonal dimensions of perceptual experience: presence (modal vs. amodal) and component (boundary vs. feature). For the present discussion, the contour/surface, shape/substance, and boundary/feature dichotomies are taken as equivalent. Notice that in their illustration of the taxonomy, Pessoa et al. did not take into account a basic heterogeneity of the two dimensions: while the first refers to mutually exclusive attributes (logically, a given perceptual entity is either modally or amodally present), this is not the case for the second dimension. In fact, the second dimension should be rather split into three levels such as boundary completion only, surface filling in only, boundary completion and surface filling in.

Such a classification is useful to evaluate the role of the minimum principle in explaining the phenomenology of AC. In the Gestalt framework, perceptual completions (both modal and amodal) are key phenomena because they reveal inner forces of organization, when outer forces are weak or absent (Koffka, 1935). For instance, in the field model of contour interpolation (Fantoni & Gerbino, 2003), amodal trajectories are interpreted as the combination of two vector fields, corresponding to two minimization tendencies: the tendency to connect segment endpoints by the shortest path (minimum distance) and the tendency to extrapolate contour segments (minimum change of direction). As regards surface properties, the illusory shrinkage of partially occluded shapes has been interpreted as a consequence of the tendency to minimize the extent of amodally represented surfaces (Kanizsa, 1972, 1975, 1979).^5^ Minimal area—one of the fundamental principles of figure/ground articulation (E. Rubin, 1915)—can also be interpreted as a tendency at the level of AC. Given a bistable pattern, the tendency of regions with a smaller extent to play the figural role could be a by-product of the minimization of the amodal ground (i.e., *ceteris paribus*, the preferred solution is the one with a smaller amodal substance).

**Figure 9.**
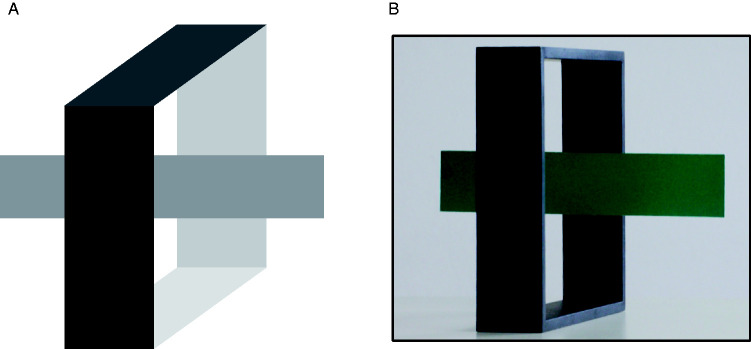
Panel A shows a pictorial display in which the depth localization of the horizontal rectangle is undeterminate, given that the display is compatible with any localization of the rectangle from just behind the front face of the open box to just in front of its back face. Most observers report a preference for seeing the rectangle just behind the front face of the open box. The photo in Panel B illustrates the dominance of depth minimization over stereoscopic cues, when observers look at a 3D object with the green horizontal lamina glued on the back face of the open box (and, therefore, abundantly detached from the front surface).

As previously reminded, Michotte et al. (1991, p. 162) included at least some aspects of 3D perception in the category of *complément amodal ‘à découvert.’* Such a suggestion has been largely ignored in the literature. However, a phenomenon interpretable in this vein was presented at the European Conference on Visual Perception in Arezzo by Gerbino and Fantoni (2007). Figure 9A shows the basic display in which a horizontally elongated rectangle could be perceived at any depth between the two vertical faces of the open box, given that interposition cues provide only partial depth information. In such conditions, a preference for perceiving the horizontal rectangle just behind the front face is observed, consistent with depth minimization (i.e., of distance from the viewpoint). The strength of such a tendency is demonstrated also by the dominance of such illusory localization in a real 3D setting (Figure 9B), where the horizontal lamina is glued to the back face of the open box, quite far apart from the front face.

## When AC Does Not Occur

Our understanding of AC could be improved not only by an adequate explanation of various types of AC phenomena (fundamentally articulated in shape vs. substance completions, as argued in the preceding section) but also by a satisfactory account of the actual nonoccurrence of AC in situations in which it could be expected, according to our best formulation of current theorizing about completion phenomena.

Two candidate nonoccurrencies have been mentioned in the subsection “Visual versus conceptual incompleteness.” They refer to the partial occlusion of a complex pattern such as a face or a superstructure such as a checkerboard. Neither a fully covered eye, in the first case, nor a fully covered component square, in the second, is amodally recovered. These cases are often explained by the dominance of local versus global cues in completion processes.^6^

**Figure 10.**
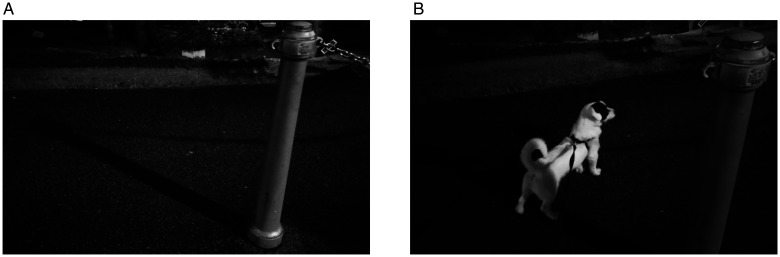
Panel A illustrates a common scene containing a casting object (a pole), its shadow cast on the sidewalk, and ample information about lighting. The cast shadow is perceived as a dark thin layer on the sidewalk, while the shadow body is not experienced perceptually. The perceptual absence of the shadow body is even more surprising when the shadow partially cast on the dog provides more information about the shape of the shadow body (Panel B).

Another possible nonoccurrence of AC has been discussed by Gerbino (2014), who argued that shadow perception is deeply affected by a (somewhat surprising) absence of AC. Cast shadows are perceived (and hence conceived by naïve observers) as flat—that is, nonvolumetric—entities. This is probably one of the most pervasive, intriguing, and neglected violations of veridicality in everyday visual perception. Cast shadows are everywhere, and often both the object casting the shadow and the projected shadow, as well as abundant visual information about the light source, are available to the observer (such as in Figure 10A). Nevertheless, the shadow is perceived as a thin layer attached to the surface on which it is cast. Metelli (1985) reminded us that a cast shadow is such as a mantle adherent on the surface of support, as in Chamisso’s *Peter Schlemihl*. Casati (2000, p. 34) remarked that “the shadow is flat: probably, it is the only non abstract object which is really 2D.”^7^ Casati and Cavanagh (2019) observed that the shadow “does not have any volume of its own” (p. 140), and that the so-called “shadow bodies” (i.e., the volumes of unilluminated space between casting objects and projection surfaces) “do not seem to enter into the perceptual processing of shadows” (p. 193) and do not have a specific name in English (p. 192).^8^

Shadow bodies are revealed in special conditions, when the atmosphere contains dust, smoke, or fog, and constitute a feature of volumetric lighting, a technique developed in cinematography and computer graphics. Under such conditions, shadow bodies become modal entities, supported by local optic information mediated by a partially reflecting atmosphere. However, as Gerbino (2014) suggested, in a perfectly transparent atmosphere (i.e., without a reflectance component) one might expect that shadow bodies were amodally present, at least when the location of the light source, the shape of the casting object, and the corresponding shape of the cast shadow are all visually available. Instead, the shadow body remains an elusive entity, even when an object partially falls into it, such as in Figure 10B.

We may ask: What are the reasons for the absence of amodal shadow bodies in ordinary conditions, given that AC processes can go beyond the fragmentary sensory evidence? Even a merely speculative answer would contribute to our understanding of limits of AC processes.

## Conclusion

AC is a popular but tricky notion. Some difficulties of the concept derive from ambiguities of the constituent terms: Amodal is used sometimes in a phenomenological sense, to mark the absence of color (when referred to vision), sometimes in a psychophysical sense, to mark the absence of a local proximal stimulus; completion can mean either complement (i.e., a simple addition to modal parts) or completion proper (i.e., something required to make proximal parts complete).

Despite such conceptual ambiguities and the vagueness of some phenomena at the interface of perception and cognition, AC provides important hints about inner forces of organization. Several AC phenomena regarding the invisible presence of either shape or substance are interpretable as manifestations of the minimum principle and contribute to a coherent theoretical framework.

Future research should elucidate the limits of AC, looking at the cases in which it does not occur, though logically expected, as well as to the ubiquitous achievements of such an enigmatic integrative process.

## Figures and Tables

**Figure A1.**
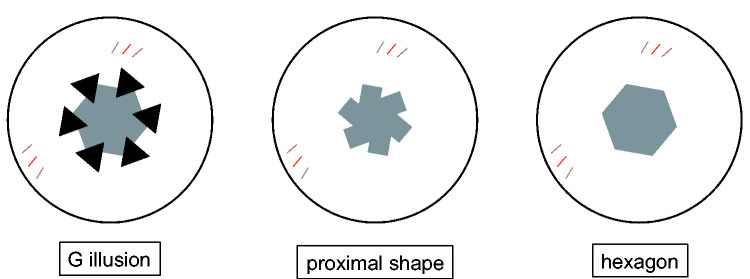
Stimuli Utilized for Collinearity Adjustments. The experimental display was a coincidentally occluded hexagon, with neither sides nor symmetry axes aligned along cardinal directions (Gerbino illusion, left). Control displays were the gray proximal shape in the absence of occluding triangles (middle) and the full regular hexagon (right). The observer adjusted the orientation of a probe made of two thin red lines (from +9° to −9°) to make it collinear with either the upper left or the lower right hexagon side (the probe for the lower right side is not shown in the figure). The orientation of both sides was 50° from the horizontal, counterclockwise. In the figure, red lines are objectively collinear with the relevant side, while gray lines mark the extreme of the ±9° range.

**Figure A2.**
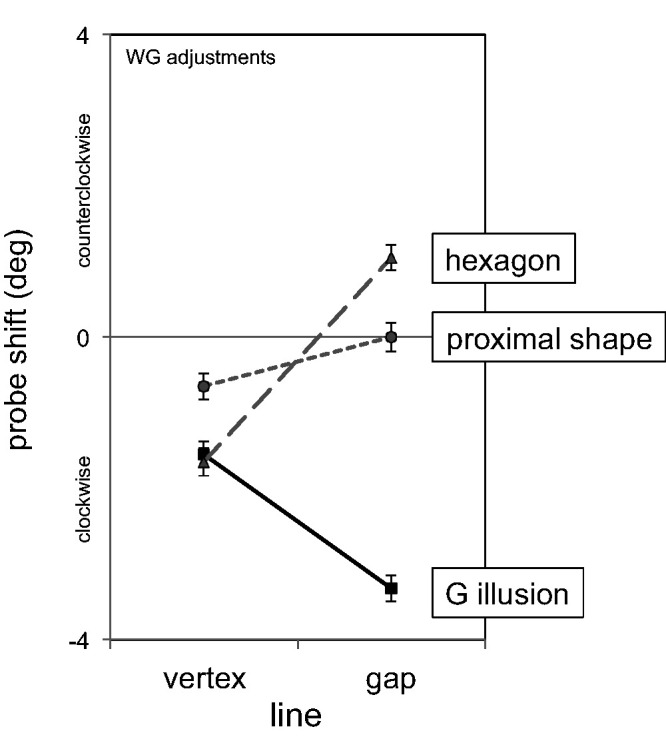
Mean Collinearity Adjustments (±1 *SEM*) for the Three Displays in Two Observation Conditions (attending to the vertex vs. gap line). Each point is the average of 40 adjustments (after pooling data from upper left and lower right sides). All relevant differences are significant at the *p* <.001 level.

## Notes

1 In Koffka’s episode, the witness is forced to admit that the book might actually cover a hole in the table where a revolver could be hidden, against the perceptual evidence of the tabletop as solid and uninterrupted also behind the book, like in its directly visible portion.

2 This translation issue has been discussed also by Scherzer and Faul (2019).

3 Take, for instance, the definition of AC provided by Nanay (2018) in his abstract and at the beginning of his introduction: “Amodal completion is the representation of those parts of the perceived object that we get no sensory stimulation from. In the case of vision, it is the representation of occluded parts of objects we see…” This definition disregards the historical fact that Michotte et al. (1964/1991) exemplified also cases of AC in the absence of occlusion.

4 For a discussion of recognition from partial information, as used by Kellman and his collaborators (Kellman, 2000; Carrigan et al., 2016), see Peta et al. (2019).

5 For alternative explanations of phenomenal shrinkage, see Vezzani (1999), Pinna and Tanca (2010), Pinna (2012), and González et al. (2005). For a similar effect in action, see Bruno and Bernardis (2002).

6 Metzger (1936/2006, Figure 140) observed that perception of a crowd lies at the interface between perception and cognition. Past experience contributes to perceiving a crowd as made of complete individuals (though only small parts are present in the proximal stimulus); but the feeling of “continuing behind” is—according to Metzger—independent of acquired knowledge.

7 Clearly, cast shadows—even when conceived as a thin layer attached to the projection surface—are neither flat nor 2D *per se*. Since they follow the spatial structure of the projection surface, they are flat and 2D only if this surface is flat and 2D. As a counterexample, take the 3D structure of a shadow cast on a staircase.

8 The shadow body does not have a specific name in Italian either.

## Appendix

The author’s data for a collinearity adjustment procedure are reported. A total of 80 adjustments for each of 3 displays illustrated in Figure A1 were made, in the following order: 40 adjustments for the upper left side of the Gerbino illusion; 40 for the corresponding side of the proximal shape; 40 for the corresponding side of the full hexagon; 40 for the lower right side of the Gerbino Illusion, and so on. No difference emerged between average adjustments for upper left and lower right sides. Hence, data from these two conditions were collapsed in Figure A2.

Every block of 40 adjustment trials included the manipulation of attention to one of the two red lines that make up the probe, defined as the “vertex line” and the “gap line” (the lower left and the upper right in Figure A1, respectively), depending on relative proximity to either the hexagon vertex included in the unoccluded portion of the hexagon side or the occluded portion of the hexagon side. As regards the Gerbino illusion, the observer first looked at the display adopting a global attitude, until the distortion was clearly experienced; then, he attended to either the vertex or the gap red line, in alternating trials, and completed the adjustment by moving the probe until the subjective collinearity with the unoccluded portion of the hexagon side was achieved. In control conditions, the probe was adjusted to make it collinear with the corresponding side of either the proximal shape or the full regular hexagon.

Mean adjustments plotted in Figure A2 indicate a clear difference between experimental and control conditions. On the whole (averaging adjustments for vertex and gap lines), the point of subjective collinearity for the Gerbino illusion was clearly shifted in the clockwise direction, corresponding to the contrast misorientation effect predicted by the approximation model, while those for the two control conditions were much closer to objective collinearity. Adjustments in the two attentional conditions differed and pointed in opposite directions for experimental and control conditions. This interaction suggests that at least a component of the distortion observed in the Gerbino illusion involves the misorientation of the unoccluded portion of the hexagon sides experienced when observers pay attention to the region of occlusion and—so to speak—try to extrapolate the unoccluded side in the direction of the coincidentally occluded vertex.
